# Mapping Quantitative Trait Loci Affecting Biochemical and Morphological Fruit Properties in Eggplant (*Solanum melongena* L.)

**DOI:** 10.3389/fpls.2016.00256

**Published:** 2016-03-04

**Authors:** Laura Toppino, Lorenzo Barchi, Roberto Lo Scalzo, Eristanna Palazzolo, Gianluca Francese, Marta Fibiani, Antonietta D'Alessandro, Vincenza Papa, Vito A. Laudicina, Leo Sabatino, Laura Pulcini, Tea Sala, Nazzareno Acciarri, Ezio Portis, Sergio Lanteri, Giuseppe Mennella, Giuseppe L. Rotino

**Affiliations:** ^1^Consiglio per la Ricerca in Agricoltura e l'Analisi dell'Economia Agraria-ORL, Unità di Ricerca per l'OrticolturaMontanaso Lombardo, Italy; ^2^Dipartimento di Scienze Agrarie, Forestali e Alimentari, Plant Genetics and Breeding, University of TurinTurin, Italy; ^3^Consiglio per la Ricerca in Agricoltura e l'Analisi dell'Economia Agraria-IAA, Unità di Ricerca per i Processi dell'Industria AgroalimentareMilano, Italy; ^4^Dipartimento di Scienze Agrarie e Forestali, Università degli Studi di PalermoPalermo, Italy; ^5^Consiglio per la Ricerca in Agricoltura e l'Analisi dell'Economia Agraria-ORT, Centro di Ricerca per l'OrticolturaPontecagnano-Faiano, Italy; ^6^Consiglio per la Ricerca in Agricoltura e l'Analisi dell'Economia Agraria-ORA, Unità di Ricerca per l'OrticolturaMonsampolo del Tronto, Italy

**Keywords:** QTL, Solanaceae, fruit quality, bioactive compounds, anthocyanins, chlorogenic acid, glycoalkaloid

## Abstract

Eggplant berries are a source of health-promoting metabolites including antioxidant and nutraceutical compounds, mainly anthocyanins and chlorogenic acid; however, they also contain some anti-nutritional compounds such as steroidal glycoalkaloids (SGA) and saponins, which are responsible for the bitter taste of the flesh and with potential toxic effects on humans. Up to now, Quantitative Trait Loci (QTL) for the metabolic content are far from being characterized in eggplant, thus hampering the application of breeding programs aimed at improving its fruit quality. Here we report on the identification of some QTL for the fruit metabolic content in an F_2_ intraspecific mapping population of 156 individuals, obtained by crossing the eggplant breeding lines “305E40” × “67/3.” The same population was previously employed for the development of a RAD-tag based linkage map and the identification of QTL associated to morphological and physiological traits. The mapping population was biochemically characterized for both fruit basic qualitative data, like dry matter, °Brix, sugars, and organic acids, as well as for health-related compounds such chlorogenic acid, (the main flesh monomeric phenol), the two peel anthocyanins [i.e., delphinidin-3-rutinoside (D3R) and delphinidin-3-(*p*- coumaroylrutinoside)-5-glucoside (nasunin)] and the two main steroidal glycoalkaloids, solasonine, and solamargine. For most of the traits, one major QTL (PVE ≥10%) was spotted and putative orthologies with other Solanaceae crops are discussed. The present results supply valuable information to eggplant breeders on the inheritance of key fruit quality traits, thus providing potential tools to assist future breeding programs.

## Introduction

Eggplant (*Solanum melongena* L.) is a member of the Solanaceae, a large plant family comprising over 3000 species including important crops such as tomato (*Solanum lycopersicum* L.), potato (*Solanum tuberosum* L.), pepper (*Capsicum annuum* L.), and tobacco (*Nicotiana tabacum* L.). Eggplant is represented by three cultivated species: *S. macrocarpon* L. and *S. aethiopicum* L., which are indigenous to a vast area of Africa and are locally cultivated, and the worldwide-cultivated *S. melongena* L. Unlike most of the other Solanaceous crops, which are native of the New World (Fukuoka et al., 2010; Albert and Chang, 2014; Hirakawa et al., 2014), eggplant has a phylogenetic uniqueness, due to its exclusive Old World origin (Lester and Hasan, 1991) located in Asia as a result of two/three separate domestication events (Daunay and Hazra, 2012; Meyer et al., 2012; Cericola et al., 2013; Knapp et al., 2013). Eggplant was introduced to the Mediterranean Basin by Muslim invaders in the Seventh-to Eighth-century CE (Daunay, 2008) and, at present, it is one of the most consumed vegetables in the world. Its global production is estimated to be around 49.5 Mt and is mainly concentrated in Asia (93% of both the world production and harvested area), with China, India, Indonesia, Iran, representing the major producers. Egypt, Turkey, and Italy are the main producers of the Mediterranean countries (FAO, 2013; http://faostat3.fao.org/browse/Q/QC/E).

Despite the economic importance of *S. melongena*, genetic mapping studies on this species have been rather limited compared to other Solanaceae crop species. The first eggplant linkage map was constructed from an interspecific (eggplant × *S. linnaeanum*) F_2_ population by Doganlar et al. (2002a) and exploited for QTL analyses of several breeding traits (Doganlar et al., 2002b; Frary et al., 2003). The same map was later on improved with the addition 115 PCR-based tomato orthologous markers (Wu et al., 2009) and used for the identification of several QTL affecting morphological traits (Frary et al., 2014). A further inter-specific map (eggplant *x S. incanum*) was recently developed by Gramazio et al. (2014) and exploited for locating polyphenol oxidase genes as well as genes involved in the chlorogenic acid biosynthetic pathway.

The first intra-specific genetic map was published by Nunome et al. (2001), and was subsequently integrated with SSR markers (Nunome et al., 2003, 2009) as well as orthologous Solanum gene sets (Fukuoka et al., 2012). Miyatake et al. (2012) spotted QTL underpinning parthenocarpy in F_2_ populations obtained by crossing one parthenocarpic with two non-parthenocarpic breeding lines; the same populations were recently employed to explore QTL for the resistance trait to *Fusarium oxysporum* (Miyatake et al., 2015) taking advantage from sequence information retrieved from the first eggplant draft genome released (Hirakawa et al., 2014). Barchi et al. (2010) developed a further intra-specific linkage map from an F_2_ population, afterwards densely populated with RAD-tag derived marker and used to characterize the genetic basis of traits associated with anthocyanin content (Barchi et al., 2012). More recently, the same map was used for detecting QTL of key horticultural traits (Portis et al., 2014). Furthermore, through a GWAs approach, the previously identified loci were validated and a number of new marker/trait associations detected (Cericola et al., 2014; Portis et al., 2015).

In the last decades the eggplant breeding goals have been mainly focused on the improvement of agronomic traits, including fruit size, weight, color, and shape (Kashyap et al., 2003; Frary et al., 2006), prickliness, yield potential and adaptation to climatic conditions (Daunay et al., 2001). Resistance or tolerance to biotic stresses have been also central breeding objectives (Rotino et al., 2014) Only recently organoleptic and nutritional properties, bioactive and anti-nutritional compounds, post-harvest and processing-related traits of eggplant fruits have been studied in a genetic/genomic perspective (Prohens et al., 2007, 2013; Gajewski et al., 2009; Prohens et al., 2013; Plazas et al., 2013; Zhang et al., 2014; Docimo et al., 2016; Lo Scalzo et al., 2016).

Eggplant berries are a source of vitamins (Grubben, 1977) and other health-promoting metabolites including anthocyanins (delphinidin glycosides) and chlorogenic acid (Stommel and Whitaker, 2003; Mennella et al., 2010) with nutraceutical and anti-oxidant properties (Cao et al., 1996; Kwon et al., 2008; Akanitapichat et al., 2010). Otherwise, they also contain some anti-nutritional compounds, like saponins and steroidal glycoalkaloids (SGA), which are responsible of the bitter taste of the flesh (Aubert et al., 1989; Sánchez-Mata et al., 2010) and with a potential toxic effects on humans. The accumulation of these metabolites in the fruit and the total amount of all these metabolites appears developmentally regulated (Mennella et al., 2012) and, although strongly affected by the environment, a wide variation among different eggplant cultivars was highlighted (Plazas et al., 2013).

To date a very limited number of studies have been aimed at identifying QTL affecting the eggplant content in bioactive and antinutrional compounds as well as other fruit quality traits (Shetty et al., 2011; Gramazio et al., 2014). Here we report the first QTL associated to the eggplant fruit content in sugars, organic acids, dry matter and soluble solid, the steroidal glycoalkaloids solamargine, the two anthocyanic pigments delphinidin-3-rutinoside (D3R) and delphinidin-3-(*p*-coumaroylrutinoside)-5-glucoside (nasunin), chlorogenic acid, as well as fruit skin color.

## Materials and methods

### Mapping population and the evaluation of phenotype

**Figure 1:**
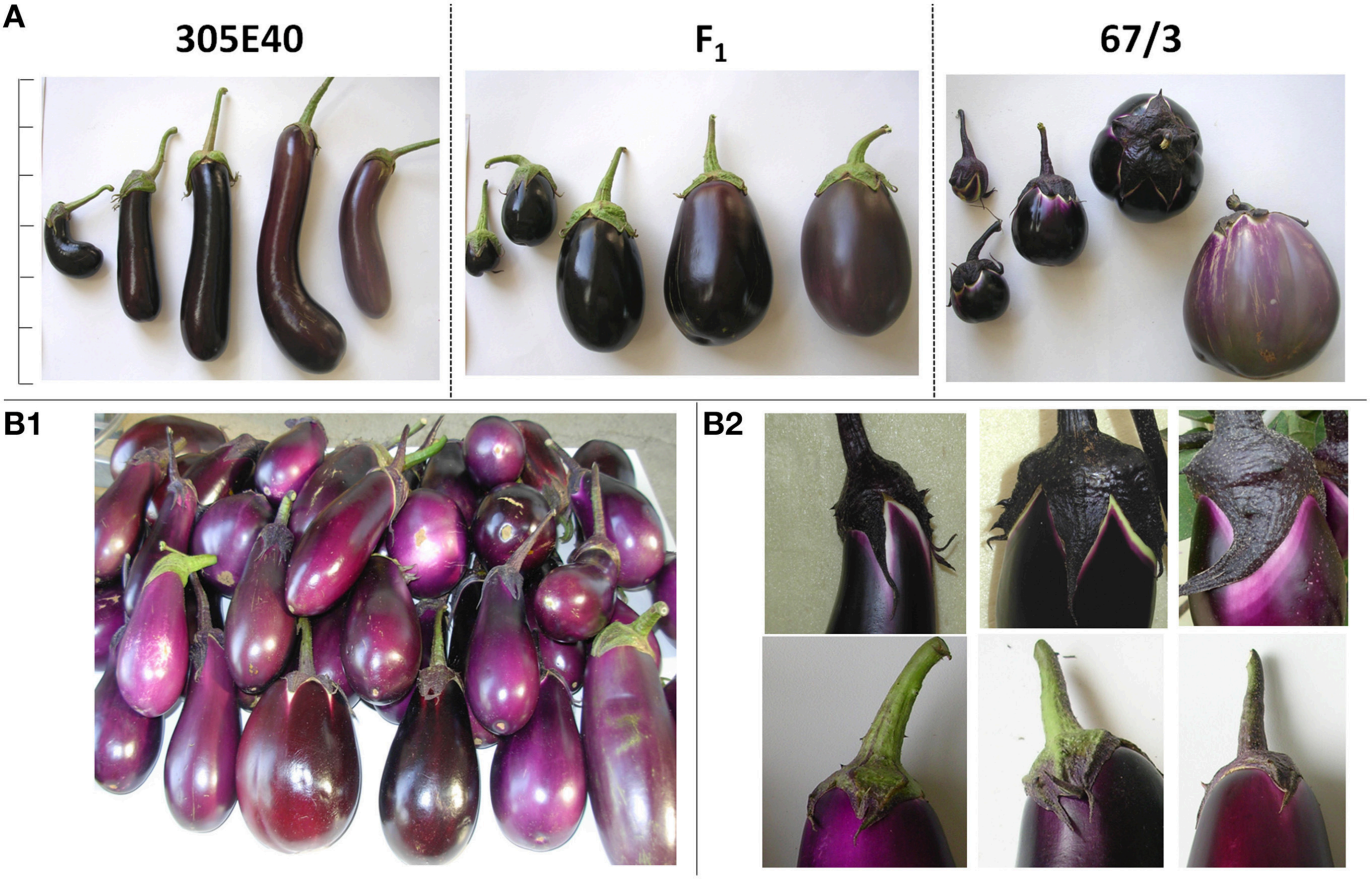
**Phenotypical characterization of the parental lines and of the F_1_ progeny. (A)** Features of the parental lines “305E40” and “67/3” and of the hybrid F_1_ at different ripening stages. For each line, from left to right are represented: stage A- (early immature, ~14 DAP), stage A (immature, ~21 DAP), stage B- (early commercial ripe, ~30 DAP), stage B (commercial ripe, ~38 DAP), stage C (physiological ripe, ~55 DAP scale bar represents 30 cm. Fruit features: **(B1)**: Detail of fruits differing for peel color. **(B2)**: Detail of fruits differing for color under and next to calyx.

A population of 156 F_2_ plants, previously obtained by crossing the two eggplant breeding lines “305E40” and “67/3,” contrasting for a wide number of key agronomic traits (Barchi et al., 2012; Portis et al., 2014) was employed (Figure 1). The double haploid female parent “305E40” was derived from an interspecific somatic hybrid *Solanum aethiopicum* gr. gilo(+)*S. melongena* cv. Dourga (Rizza et al., 2002), which was repeatedly backcrossed with the recurrent lines DR2 and Tal1/1, prior to selfing and anther culture. The female parent carries the resistance locus *Rfo-sa1* conferring resistance to the soil-borne fungus *F. oxysporum* f. sp. *melongenae* (Toppino et al., 2008). “305E40” produces pink flowers and long, highly pigmented dark purple fruits characterized by the presence of the anthocyanin D3R as well as a higher glycoalkaloids and organic acid content than the line “67/3.” The latter is the result of an F_8_ selection from the intra-specific cross cv. “Purpura” × cv. “CIN2,” displays higher anthocyanic pigmentation than “305E40” in leaves and stems, and produces violet flowers and round, violet colored fruits with white peel color both under and next to calyx. The fruits are characterized by the presence of the anthocyanin nasunin in the peel, higher soluble solids, sugars, and chlorogenic acid content in the flesh with respect to “305E40”.

The mapping population was field grown, along with both parents and the F_1_ hybrid, at two sites, namely ML (Montanaso Lombardo 45°20′N; 9°26′E) and MT (Monsampolo del Tronto 42°53′N; 13°47′E). Each F_2_ individual was replicated by establishing vegetative cuttings. To this end, cuttings were obtained both from apical and lateral shoots, by using a sharp scalpel, from greenhouse-grown plantlets at the 6–8 leaves stage. To stimulate rooting the cut surface of the cutting was immersed for 10 min in a solution containing 2 mg/l of IBA (indol 3 butyric acid) and 5 mg/l of NAA (α-Naphthaleneacetic acid) buffered to pH 6.0 using 500 mg/l of MES [2-(N-morpholino)ethanesulfonic acid] and then grown in small pot (9 × 9 cm) filled with peat and perlite (70:30 v/v). The pots were maintained in the shadow under high humidity to preserve leaf viability until rooting. This procedure was repeated three times to clone the entire F_2_ population. At both sites, the material was arranged as a set of two randomized complete blocks with two replicate plants per entry per block. The traits scored are detailed in Table 1.

**Table 1:** **List of the traits (unit of measurement for all the biochemical compounds is mg/100 dw) analyzed and their code, means, standard deviations (SD), coefficients of variation (cv), and broad sense heritability for the traits**.

**Trait**	**Code**	**Env**	**Parents mean ± SD**		**F_1_ mean ±SD**	**F_2_ population**	**cv**	**Skewness**	***SE***	**Kurtosis**	***SE***	**Heritability**	**Trasgressive respect “305E40”**	**Trasgressive respect “67/3”**
			**“305E40”**	**“67/3”**										
Fruit color	*Frucol*	ML	14	24	18	22.0 ± 4.7	0.21	−1.20	0.10	0.65	0.20	0.98	10	23
		MT	14	23.7 ± 0.5	18	21.6 ± 5.1	0.23	−1.08	0.10	0.37	0.20	0.97	9	26
Undercalyx color	*Undcal*	ML	12.6 ± 0.5	0	17	13.4 ± 10.2	0.76	−0.18	0.10	−1.50	0.20	0.97	80	0
		MT	12.5 ± 0.5	0	16.5 ± 0.7	13.9 ± 10.2	0.73	−0.35	0.10	−1.54	0.20	0.98	83	0
Peel next calyx color	*Pncc*	ML	0	2	1	0.8 ± 0.9	1.13	0.47	0.10	−1.53	0.20	0.98	0	0
		MT	0	2	0.5	0.9 ± 0.8	0.88	0.18	0.10	−1.47	0.20	0.97	0	0
D3R	*D3R*	ML	790 ± 12	0.0	0.00	224 ± 509	2.27	2.67	0.20	6.60	0.40	0.99	15	0
		MT	764 ± 9	0.0	0.00	215 ± 483	2.24	3.44	0.19	14.77	0.39	0.98	15	0
Nasunin	*Nas*	ML	0	301 ± 26	295 ± 10	202 ± 192	0.95	2.49	0.20	8.47	0.40	0.97	0	10
		MT	0	303 ± 7	300 ± 5	221 ± 209	0.94	2.26	0.19	6.68	0.39	0.96	0	15
Dry matter (%)	*DM*	ML	8.1 ± 0.9	9.3 ± 0.7	7.7 ± 0.2	8.2 ± 1.0	0.11	1.06	0.19	2.87	0.39	0.64	1	0
		MT	12.5 ± 1.3	10.2 ± 0.2	14.7 ± 0.1	10.8 ± 2.6	0.23	2.03	0.19	6.47	0.39	0.51	3	54
Soluble solid content	*SSC*	ML	40.4 ± 1.1	50.7 ± 3.0	49.8 ± 1.6	42.7 ± 4.7	0.11	0.04	0.19	14.62	0.39	0.41	13	0
		MT	48.1 ± 3.6	55.4 ± 2.1	49.9 ± 1.9	43.7 ± 5.4	0.12	0.28	0.19	9.07	0.39	0.43	30	0
Solamargine	*SM*	ML	6.2 ± 0.6	6.2 ± 0.2	11.7 ± 1.1	4.5 ± 4.0	0.88	1.84	0.19	4.16	0.39	0.92	31	117
		MT	3.8 ± 0.7	1.7 ± 0.1	1.9 ± 0.1	5.8 ± 3.9	0.68	1.90	0.19	7.15	0.39	0.89	78	12
Solasonine	*SS*	ML	42.1 ± 2.6	19.9 ± 1.1	28.8 ± 0.8	24.2 ± 7.7	0.32	0.48	0.19	−0.31	0.39	0.85	0	36
		MT	17.1 ± 1.8	7.1 ± 0.5	27.7 ± 2.5	34.6 ± 12.7	0.37	1.09	0.19	1.00	0.39	0.74	145	0
Fructose	*Fru*	ML	13241 ± 2960	17973 ± 6015	18012 ± 3847	19144 ± 5060	0.26	0.74	0.20	3.89	0.39	0.82	0	3
		MT	14757 ± 252	20514 ± 283	21539 ± 1202	20023 ± 5179	0.25	0.41	0.20	0.97	0.39	0.80	10	60
Glucose	*Glc*	ML	15000 ± 1018	18611 ± 4203	18536 ± 1811	18019 ± 5478	0.3	1.18	0.20	4.07	0.39	0.86	15	6
		MT	21386 ± 1315	22259 ± 1852	25531 ± 2815	12954 ± 9880	0.76	12.98	0.20	205.90	0.39	0.67	150	0
Sucrose	*Suc*	ML	1345 ± 134	943 ± 295	1124 ± 209	2104 ± 1078	0.51	1.97	0.20	8.25	0.39	0.88	0	106
		MT	516 ± 41	674 ± 23	1785 ± 123	2371 ± 1601	0.67	2.51	0.20	8.39	0.39	0.77	0	153
Quinic acid	*QA*	ML	3469 ± 798	1814 ± 473	2230 ± 232	4136 ± 981	0.23	2.97	0.19	19.43	0.39	0.77	20	0
		MT	53658 ± 3428	2174 ± 111	1442 ± 166	3133 ± 1015	0.32	5.85	0.19	55.05	0.39	0.65	1	4
Citric acid	*CA*	ML	8648 ± 3098	268 ± 151	64 ± 60	1201 ± 2694	2.24	3.15	0.19	11.59	0.39	0.80	31	0
		MT	8778 ± 788	823 ± 82	119 ± 9	553 ± 1015	1.84	2.40	0.19	6.33	0.39	0.73	32	116
Ox alic acid	*OA*	ML	29768 ± 1702	3871 ± 2390	830 ± 3	1423 ± 405	0.28	0.52	0.19	0.06	0.39	0.69	0	0
		MT	5263 ± 507	4145 ± 311	389 ± 15	1849 ± 343	0.19	0.54	0.19	2.17	0.39	0.69	0	156
Skichimic acid	*SA*	ML	1061 ± 175	566 ± 16	830 ± 3	904 ± 180	0.2	1.13	0.19	2.39	0.39	0.68	2	0
		MT	1290 ± 154	663 ± 56	389 ± 15	599 ± 116	0.19	0.58	0.19	0.90	0.39	0.71	0	54
Chlorogenic acid	*CGA*	ML	2085 ± 42	2432 ± 16	2105 ± 20	1801 ± 328	0.18	1.07	0.19	2.13	0.39	0.90	126	6
		MT	2056 ± 45	2460 ± 66	2161 ± 48	1789 ± 443	0.25	0.39	0.20	0.28	0.39	0.92	104	6

Significant mean difference among parental values (Wilcoxon test) is reported *p < 0.05. Skewness and kurtosis [with their standard errors (SE)] are also listed.

### Phenotypic trait evaluation

The fruit color was evaluated in two representative fruits per plant, collected at the commercial stage (stage B, as in Mennella et al., 2012; Figure 1) between the first and fourth harvest. A discrete phenotypic scale was used to characterize the skin color both as a whole and under the calyx, taking into consideration both the tonality (0 = white, 5 = reddish, 10 = purple, 15 = intermediate, 20 = violet, 25 = brilliant lavender) and the intensity (+0 = whitish, +1 = light +2 = normal, +3 = dark, +4 = blackish). For the presence of a lighter peel edge next to calyx, a scale from 0 to 2 (0 = absent, 1 = intermediate, 2 = present) was utilized.

### Biochemical trait evaluation

Samplings for biochemical analyses were carried out at two fruit ripening stages, unripe [A, approximately 21 DAF (days after flowering), and commercial (B, approximately 38 DAF)]. According to Mennella et al. (2012), fruits at the stage A are still actively growing, close to half of the final size, with glossy skin color, calyx, and peduncle quite tender and flexible, flesh still soft and greenish or white and seeds having not reached final size and displaying a white tegument. Besides, fruits at the stage B have reached their commercial final size, with brighter or less brilliant skin (and eventually little dull in some lines); the flesh is less greenish and, in the 305E40 like-typology shows the characteristic green ring next to the skin (absent in “67/3”), seeds have almost reached their final size but are still immature.

The developing stages A was chosen as reference for the flesh biochemical characterization because, in a preliminary study, it was the stage in which the parental lines showed the highest significant differences with regard to the content of the majority of the compounds in study (Supplementary Table 1). Only for the D3R and nasunin content, the peel from fruits at developing stage B were analyzed.

The experimental sample was constituted by portions obtained from 5−8 fruits, sampled in duplicate. For fruits at stage A, fruit cubes were obtained within 2 h after harvest; while from fruits at stage B, peel slices of about 4 cm^2^ were collected from the same fruit analyzed for skin color. All the samples were immediately frozen in liquid N_2_ and lyophilized.

The fresh weight of all the samples and the correspondent weight at the end of freeze-drying process were detected for dry matter (DM) value calculation. The freeze-dried tissues were powdered and held at −80°C. All results were referred to as dry weight (dw).

Peel anthocyanins, glycoalkaloids (solamargine and solasonine) and phenolic acids of the fruit flesh were extracted and analyzed according to Mennella et al. (2012). The extraction and the analysis of anthocyanins was carried out on 200 mg of lyophilized and powdered peel, diluted in 10 mL of methanol containing 3% trifluoroacetic acid (TFA). RP-HPLC analysis was performed through a Waters E-Alliance HPLC system constituted by a 2695 separations module with quaternary pump, autosampler, and a 2996 photodiode array detector; data were acquired and analyzed with Waters Empower software on a PC. The chromatographic separations were performed at a flow rate of 0.8 mL/min and at 0.1 AUFS. Purified D3R (Polyphenols Laboratories AS, Sandnes, Norway) was used as external standard in RP-HPLC analyses. As for nasunin quantification, a partially purified standard was used according to Lo Scalzo et al. (2010). The results were expressed as mg/100 g of peel dw; the limit of detection was 1.3 mg/100 g of dw.

The extraction of chlorogenic acid (CGA) started from 200 mg of lyophilized flesh. The quantification carried out after a RP-HPLC separation, was based on absorbance at 325 nm relative to the sesamol internal standard and an external standard of authentic CGA (Sigma-Aldrich, St. Louis, MO). The results were expressed as mg/100 g of dw. Soluble solid content (SSC) was measured on the centrifuged extract of 300 mg of eggplant powder with 10 mL of 1 mM HCl (5 min at 25000 × g at room temperature), and it was expressed as percent substance on dw.

Glycoalkaloids, solamargine, and solasonine, were extracted from 0.5 g samples of lyophilized and powdered flesh tissue by 95% ethanol. The analyses were performed by means of RP-HPLC using partially purified solasonine and solamargine as the external standards. The data were expressed as mg/100 g dw; the limit of detection was 0.03 mg/100 g of dw.

Organic acids and simple sugars content of eggplant extracts were measured by HPLC, slightly modifying the methods proposed and validated by Caccamo et al. (1986) and by López-Tamames et al. (1996). The extracts were the same used for SRR determination (see above).

As for organic acids, the separation was carried out on an Inertsil ODS-3 column 5 μm of particle diameter and 0.46 × 25 cm dimension. The elution was carried out at 30°C with H_3_PO_4_ 0.02 M in milli-Q water as mobile phase at 0.6 mL/min. The detection was spectrophotometrically made at 214 nm. The extracts from eggplant (3 mL) were filtered through 0.45 μm PTFE filters and 3-fold diluted with the mobile phase before injection (20 μL).

Oxalic, quinic, shikimic, and citric acid solutions were used as external standards (retention times 5.4, 6.7, 8.8, and 12.7 min, respectively), and the results were expressed as mg/100 g dw.

Sucrose, glucose and fructose were separated with a CarboSep Coregel 87C carbohydrate column with a 0.78 × 30 cm bed packed with a cation-exchange resin in the Ca^2+^ ionic form. The mobile phase was milli-Q water at 0.7 mL/min, the elution was performed at 85°C, and the signals were revealed by a refractive index detector. Samples were five-fold diluted with mobile phase before injection (20 μL). Sucrose, glucose and fructose solutions were used as external standards (retention times 9.0, 10.1, and 12.4 min, respectively) and the results were expressed as mg/100 g dw.

### Statistical analyses and QTL detection

Statistical analyses were performed using R software (R Development Core Team, 2009). A conventional analysis of variance was applied to estimate genotype and environment effects based on the linear model *Y*_i__j_ = μ + *g*_i_ + *b*_j_ + *e*_ij_, where μ, *g, b*, and *e* represent, respectively, the overall mean, the genotypic effect, the block effect and the error. Broad-sense heritability values were given by $\sigma_{G}^{2}$/([$\sigma_{G}^{2}$ + $\sigma_{\text{E}}^{2}$]/n), where $\sigma_{\text{G}}^{2}$ represented the genetic variance, $\sigma_{\text{E}}^{2}$ the residual variance and n the number of blocks. Correlations between traits were estimated using the Spearman coefficient, and normality, kurtosis and skewness were assessed with the Shapiro-Wilks test (α = 0.05). Segregation was considered as transgressive when at least one F_2_ individual recorded a trait value higher or lower by at least two standard deviations than the higher or lower scoring parental line. QTL detection was based on the Barchi et al. (2012) map, constituted of 415 markers (339 SNPs, 2 HRMs, 3 CAPSs, 11 RFLPs, 33 SSRs, and 27 COSII) and spanning 1390 cM. Putative QTL location was determined by both interval (Lander and Botstein, 1989) and MQM (Jansen, 1993; Jansen and Stam, 1994) mapping, as implemented in MapQTL v5 software (Van Ooijen, 2004). QTL were initially identified using interval mapping, after which one linked marker per putative QTL was treated as a co-factor in the approximate multiple QTL model. Co-factor selection and MQM analysis were repeated until no new QTL could be identified. LOD thresholds for declaring a QTL to be significant at the 5% genome-wide probability level were established empirically by applying 1000 permutations per trait (Churchill and Doerge, 1994). Additive and dominance genetic effects, as well as the percentage of the phenotypic variance explained (PVE) by each QTL were obtained from the final multiple QTL model. The program QTLNetwork 2.1 (Yang et al., 2007) was used to analyze each set of environment's data separately to identify epistasis, and was then extended across both environments to identify any QTL x environment interactions present. QTL effects were estimated on the basis of the Markov Chain Monte Carlo (MCMC) method. A type I error level of 0.05 was applied. The genome scan employed a 10 cM window and a 1 cM walk speed. Critical F values were obtained by 1000 permutations and a threshold of 0.05 was applied to assign significance to a QTL or to an epistatic effect. Individual QTL were prefixed by a trait abbreviation, followed by the relevant chromosome designation, and were suffixed as “a” or “b” where more than one QTL mapped to a single linkage group; ML or MT was added as a suffix where the QTL was expressed in a site-specific manner. Epistatic effects were indicated by adding “^*^” to the label of a major established QTL, while “ep” was added to a newly detected QTL. MapChart v2.1 software (Voorrips, 2002) was draw the resulting maps. Syntenic regions of the tomato genome (sequence build 2.50; http://solgenomics.net/organism/Solanum_lycopersicum/genome) were accessed to identify candidate genes co-localizing with the eggplant QTL. Initial searches were conducted using 20-kb sections and, for sections of interest, additional searches were performed using 10 kb sections. Putative tomato orthologs of the eggplant genes were identified by Blast search in the tomato gene indices at DFCI.

## Results

### Phenotypic variation and inter-trait correlations

Trait codes, their values and broad sense heritability are shown in Table 1. The parental lines significantly differ each other for most of the traits in study at both sites (Table 1). Compared to “67/3” plants, “305E40” produces purple dark fruits reaching a score of 14 (10+4, see methods), with the same peel color under (*Undcal*) and next (*Pncc*) to the calyx, contains exclusively the D3R pigment and has a greater organic acids content than the line “67/3”. On the contrary, the latter is characterized by a dark/blackish violet color rated at 23–24 (20+3−4) given by the exclusive presence of the nasunin pigment, and absence of pigmentation under calyx, together with a higher amount of sugars and chlorogenic acid. At both sites, the F_1_ hybrid was intermediate for the fruit color (rating 18 given probably by the presence of both anthocyanin nasunin and green ring in the flesh next to the peel), the content of soluble solid, solasonine, and chlorogenic acid (Table 1). For several traits, F_1_ performance was significantly superior to the better performing parent, as for *Undcal* (both environments), *DM* (MT), *SM* (ML), and sugar contents; on the contrary, as an example, *CA* and *QA* (MT) and *DM* (ML) contents were lower to the worst performing parent. In the F_2_ progeny transgressive segregation (as calculated from the raw phenotypic data (Table 1) was observed for several traits. As an example, the traits *Frucol* (23 plants), *SM* (117 plants), *Suc* (106 plants) compared to “67/3” in ML and *Undcal* (80 plants), and *CA* (31 plants) with respect to “305E40.” In MT, transgressive phenotypes were found, among the others, for *Undcal* (83 plants) and *SS* (145 plants) compared to “305E40” and for *DM* (54 plants), *Suc* (153 plants), and *CA* (116 plants) toward “67/3” parent. The broad sense heritability values were generally uniform between ML and MT. The range was from 0.41 (*SSC* at ML) to 0.99 (*D3R* at ML) (Table 1). Significant inter-trait correlations (*p* < 0.05) were detected both within and across sites (Table 2). In both ML and MT, *Frucol* was positively correlated with *Undcal* and *Nas* and negatively correlated with *D3R, Undcal* was negatively correlated with *Pncc*, and *Fru* was positively correlated with *Glc*. Finally *QA, SA*, and *OA* were positively correlated between each other and negatively correlated with *CA*. The significant correlations across sites ranged from + 0.224 for *SA* to + 0.926 for *Undcal*.

**Table 2:** **Inter-trait Spearman correlations assessed in the mapping population**.

		**Frucol**	**Undcal**	**Pncc**	**DM**	**SSC**	**D3R**	**Nas**	**SM**	**SS**	**Fru**	**Glc**	**Suc**	**QA**	**OA**	**CA**	**SA**	**CGA**
Frucol	ML	0.742^**^	0.428^**^	−0.058	0.038	−0.037	−0.373^**^	0.355^**^	−0.054	−0.128	−0.129	−0.063	0	0.053	−0.115	0.107	0.015	0.007
Frucol	MT		0.512^**^	−0.046	−0.002	0.019	−0.516^**^	0.570^**^	−0.044	−0.161^*^	−0.061	−0.097	−0.042	0.133	−0.043	−0.031	0.09	−0.047
Undcal	ML		0.926^**^	−0.676^**^	0.072	0.058	−0.180^*^	0.204^*^	−0.059	0.067	−0.021	−0.022	−0.091	0.055	−0.045	0.126	0.01	−0.051
Undcal	MT			−0.721^**^	0.08	0.002	−0.205^*^	0.320^**^	−0.085	−0.151	0.06	0.013	0.041	0.192^*^	−0.089	−0.003	0.071	−0.067
Pncc	ML			0.832^**^	−0.1	−0.111	−0.088	0.043	0.085	0.002	−0.194^*^	−0.151	0.082	−0.058	−0.009	0.019	0.058	−0.058
Pncc	MT				−0.063	−0.055	−0.049	−0.179^*^	0.139	0.099	−0.154	−0.12	−0.064	−0.115	0.074	−0.005	0.068	0.016
DM	ML				0.179^*^	−0.211^**^	0.13	0.115	0.006	0.105	0.156	0.185^*^	−0.051	0.11	−0.241^**^	0.019	0.097	0.182^*^
DM	MT					−0.183^*^	−0.024	−0.099	0.003	0.06	−0.051	−0.109	−0.046	−0.063	−0.224^**^	0.136	−0.021	0.139
SSC	ML					0.161^*^	0.112	−0.156	0.043	0.124	0.071	0.039	−0.066	0.091	0.088	0.07	0.081	−0.066
SSC	MT						0.133	0.077	−0.175^*^	0.04	0.231^**^	0.249^**^	0.208^**^	0.032	0.177^*^	−0.147	−0.043	−0.273^**^
D3R	ML						0.569^**^	−0.359^**^	−0.001	0.156	0.112	0.164^*^	−0.069	0.107	0.019	−0.12	0.048	0.038
D3R	MT							−0.339^**^	0.016	0.222^**^	0.095	0.137	0.107	−0.02	0.091	−0.064	−0.024	−0.04
Nas	ML							0.626^**^	−0.088	−0.107	−0.065	−0.095	−0.084	−0.049	−0.019	0.143	0.048	0.124
Nas	MT								−0.068	−0.122	0.051	0.064	−0.022	0.163^*^	0.1	−0.121	0.038	−0.051
SM	ML								0.443^**^	0.342^**^	0.209^**^	0.219^**^	0.086	−0.076	0.05	−0.024	−0.037	−0.093
SM	MT									0.283^**^	−0.064	−0.101	−0.077	0.076	−0.105	−0.105	0.093	0.216^**^
SS	ML									0.14	0.13	0.098	0.014	−0.032	−0.092	0.111	−0.08	−0.153
SS	MT										0.043	0.009	0.048	−0.113	−0.077	0.07	−0.124	0.076
Fru	ML										−0.091	0.857^**^	0.036	−0.061	−0.071	0.042	−0.04	0.032
Fru	MT											0.852^**^	0.171^*^	−0.164^*^	0.145	0.192^*^	−0.242^**^	−0.209^**^
Glc	ML											−0.093	0.003	−0.031	−0.073	−0.004	−0.012	−0.004
Glc	MT												0.066	−0.113	0.172^*^	0.173^*^	−0.136	−0.286^**^
Suc	ML												0.068	−0.166^*^	0.052	−0.005	−0.089	−0.172^*^
Suc	MT													−0.15	−0.047	−0.075	−0.091	0.026
QA	ML													0.139	0.388^**^	−0.392^**^	0.757^**^	0.134
QA	MT														0.232^**^	−0.439^**^	0.692^**^	0.122
OA	ML														0.289^**^	−0.566^**^	0.434^**^	0.027
OA	MT															−0.028	0.329^**^	−0.147
CA	ML															−0.058	−0.267^**^	−0.017
CA	MT																−0.184^*^	−0.064
SA	ML																0.224^**^	0.145
SA	MT																	−0.008
CGA	ML																	0.276^**^
CGA	MT																	

The values in diagonal represent correlations of the same trait between the two environments. Correlations are significant

* p < 0.05;

** *p < 0.01*.

### Identification of QTL clusters

**Figure 2:**
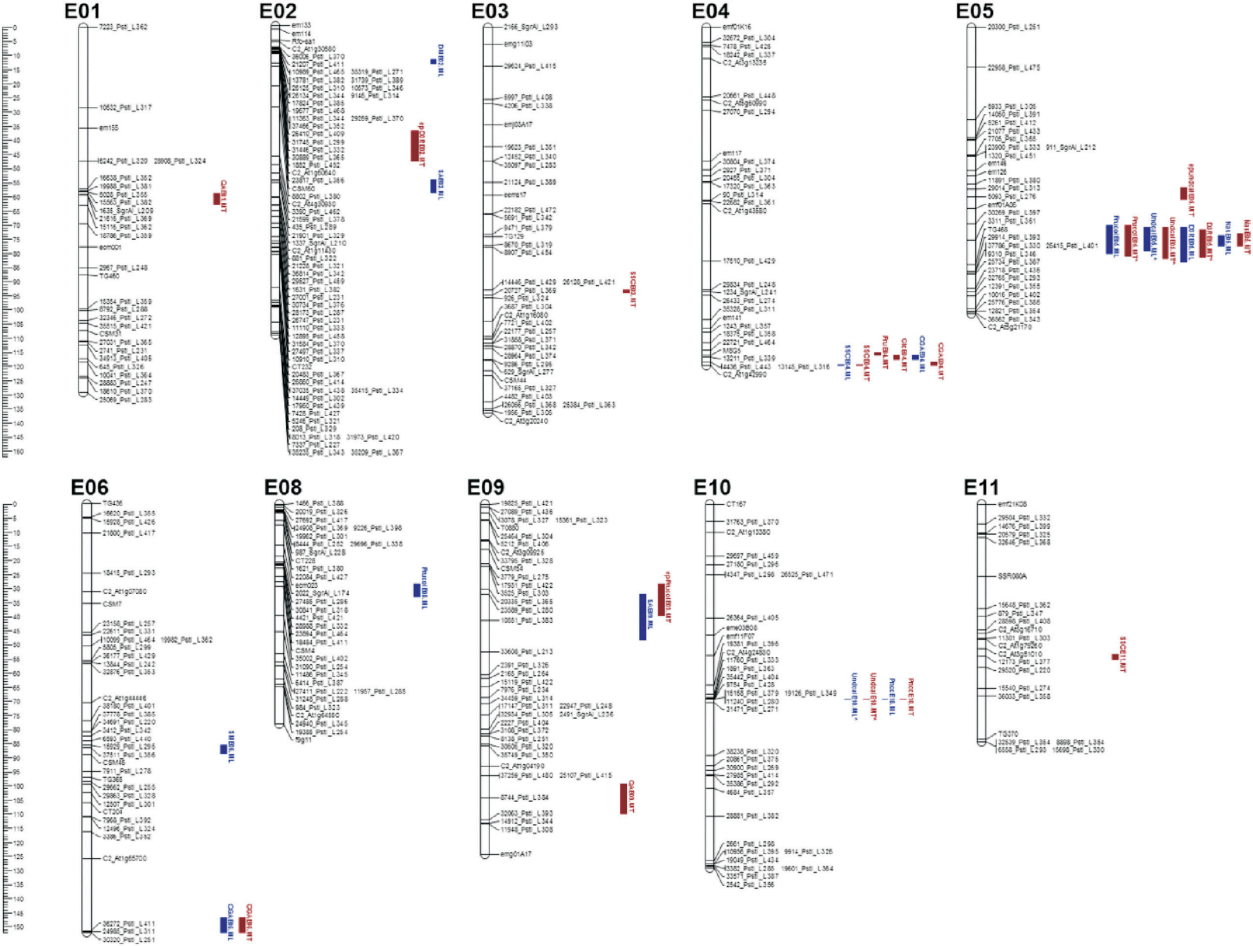
**Eggplant linkage map with graphical representation of the QTL detected**. Marker names are shown to the *right* of each chromosome, with map distances (in cM) shown on the left. Map positions of the QTL identified on each chromosome (or LG) are also given on the right. The length of the vertical bars represents the confidence interval of the QTL (LODmax^−1^ interval). QTL shown in *blue* were detected only at ML, and those in *red* only at MT. Epistatic interacting QTL are indicated by the prefix ^ where the QTL was recognized by MapQTL software.

In all, 29 QTL (of which 22 explained at least 10% of the PVE, hereafter referred to as “major” QTL) were identified and mapped onto 10 of the 12 eggplant chromosomes (Table 3, Figure 2), with the exclusion of E07 and E12. At ML and MT a set of 14 (10 major) and 15 (12 major) QTL were identified, respectively. Fourteen major QTL were uncovered at both sites, while three were exclusively detected in ML and four only in MT; finally, one was a major QTL in one of the sites and a minor one in the other. The genomic locations of QTL are shown in Figure 2. Clustering of the QTL was found in chromosomes E04, E05, and E10. The high inter-trait correlations (positive or negative) between the traits falling in QTL clusters on chromosome E05 and E10 (Table 2) suggest that they reflect either a set of closely linked loci or, more likely, a single pleiotropic locus. In the cluster on chromosome E05, the QTL were associated with fruit and under calyx color, as well as with *D3R* and *Nas* content. The other cluster which includes QTL for *Undcal* and *Pncc* was spotted on chromosome E10.

**Table 3:** **QTL detected in the mapping population**.

**Trait code**	**Chr**	**Montanaso Lombardo (ML)**									**Monsampolo del Tronto (MT)**								
**GW**	**QTL**	**Position**	**Locus**	**CI**	**LOD**	**PVE**	**A**	**D**	**GW**	**QTL**	**Position**	**Locus**	**CI**	**LOD**	**PVE**	**A**	**D**
Fruit color	5	4	FrucolE05.ML	75.304	3311_PstI_L361	70–80	32.01	56.3	−4.274	4.099	3.7	frucolE05.MT^*^	75.304	3311_PstI_L361	70−81	40.62	69.9	−5.116	5.127
	8		FrucolE08.ML	29.19	35002_PstI_L402	28.5−33	4.88	5.5	−1.138	−1.236									
Undercalyx color	5	3.8	UndcalE05.ML^*^	75.304	3311_PstI_L361	70.7−79.3	20.6	13.8	−4.339	4.958	3.9	UndcalE05.MT^*^	75.304	3311_PstI_L361	70.7−82	27.4	13.8	−4.547	4.658
	10		UndcalE10.ML	69.39	15158_PstI_L379	69.2−69.4	58.76	77	10.129	9.061		UndcalE10.MT^*^	69.39	15158_PstI_L379	69.2−69.4	72.35	82.5	10.901	8.810
Peel next to calyx color	10	3.8	PnccE10.ML	69.39	15158_PstI_L379	69.2−69.4	68.92	86.9	−0.962	−0.618	3.7	PnccE10.MT	69.39	15158_PstI_L379	69.2−69.4	58.7	82.3	−0.885	−0.456
Dry matter (%)	2	3.9	DME02.ML	13.861	C2_At1g60640	12.5−14	4.27	11.9	0.221	−0.414									
Soluble solid content	3										3.7	SSCE03.MT	92.972	26128_PstI_L421	92.9−93.9	6.65	14.7	1.839	−0.096
	4	3.7	SSCE04.ML	119.48	C2_At1g42990	119.3−119.5	3.85	10.7	−1.267	0.615		SSCE04.MT	119.478	C2_At1g42990	119.3−119.5	4.6	10.1	−1.386	0.555
	11											SSCE11.MT	53.995	12173_PstI_L377	53.3−55	3.72	7.8	0.254	−1.919
D3R	5	4.7	D3RE05.ML	75.304	3311_PstI_L361	70.7−83	21.94	49.7	447.367	−393.267	4.2	D3RE05.MT^*^	75.304	3311_PstI_L361	71.7−81.3	24.73	52	424.258	−389.531
Nasunine	5	4.2	NasE05.ML	75.304	3311_PstI_L361	73.7−77.3	10.48	28	−123.507	120.662	4.4	NasE05.MT	75.304	3311_PstI_L361	72.7−77.3	11.22	28.4	−133.158	130.719
Solamargine	6	3.9	SME06.ML	85.77	15929_PstI_L295	85.7−88.6	5.02	13.9	−2.247	−0.343									
Fructose	4	3.7									3.8	FruE04.MT	115.985	M8G5	115−116	3.79	10.7	−1675.61	1331.67
Glucose	4	3.8									3.8	GlcE04.MT	116.641	13211_PstI_L339	115.9−117.6	3.85	10.7	−862.24	1582.87
Quinic acid	1	3.5									3.1	QAE01.MT	59.021	1635_SgrAI_L209	58.7−62.5	6.63	16.4	−1161.18	−1238.28
	9											QAE09.MT	104.057	8744_PstI_L384	99.2−110	3.75	8.9	−101.98	589.527
Shikimic acid	2	3.5	SAE02.ML	58.272	21901_PstI_L329	57.6−62.2	4.67	12.1	97.214	7.385									
	9		SAE09.ML	41.331	10551_PstI_L383	32−48.1	3.56	8.2	70.602	12.947									
Chlorogenic acid	4	3.8	CGAE04.ML	116.64	13211_PstI_L339	116−117.6	2.78	7.3	113.614	−59.587	3.8	CGAE04.MT	119.478	C2_At1g42990	118.4−119.48	4.12	9.5	184.736	−45.127

For each trait the genome-wide thresholds (GW) at p = 0.05 (as determined from 1000 permutations) is indicated. The closest mapping marker to each QTL and which parent contributed positively to the trait are indicated, along with the value of the QTL, the confidence interval (CI), the LOD, the percentage of variation explained (PVE), and the additive (A)/dominance (D) contribution. Epistatic interacting QTL are indicated by the prefix

* *where the QTL was recognized by MapQTL software*.

### QTL affecting fruit color traits in eggplant

#### Fruit color (frucol)

Two *Frucol* QTL were mapped in ML and one in MT. A particularly large effect QTL (*FrucolE05*) explained 56.3% in ML and 69.9% in MT of the PVE and mapped to the same E5 region of QTL for *Undcal, D3R*, and *Nas*. A further minor QTL (*FrucolE08*) was detected only in ML and explained 5.5% of the PVE. The allele for the violet color (violet vs. purple) derived from “67/3.”

#### Under calyx color (undcal)

Two major QTL were detected in both environments and located in E05 and E10. The largest effect locus (*UndcalE10*) explained 77% of the PVE in ML and 82.5% in MT; the second major QTL (*UndcalE05*) explained 13.8% of the PVE at both the sites. For *UndcalE05*, the allele conferring the presence of under calyx pigmentation derives from “305E40” while absence of pigmentation comes from “67/3.”

#### Peel next to calyx color (Pncc)

*One* major QTL at locus *PnccE10* was found in both the environments, with a PVE of 86.9% and 82.3% in ML and MT, respectively. The segment containing this E10 locus also influenced the major QTL for *Undcal*. The positive allele (presence of a lighter peel-next to calyx color vs. no different color) was contributed by “67/3.”

### QTL determining basic qualitative traits of fruit

#### D3R (D3R)

*One* QTL (*D3RE05)* was detected in both ML and MT, mapping to E05 and explaining 49.7% and 52% of the PVE in ML and MT respectively. The positive allele was inherited from “305E40.”

#### Nasunin (Nas)

*One* QTL (*NasE05)* was detected in both ML and MT, mapping to E05 and explaining 28% and 28.4% of the PVE in ML and MT, respectively. The positive allele was inherited from “67/3.”

#### Dry matter (DM)

The major QTL *DME02* was detected only in ML environment, explaining 11.9% of the PVE. The positive allele was transmitted from “305E40.”

#### Soluble solid content (SSC)

*One SSC* QTL was mapped in ML and three in MT. The major QTL *SSCE04* was detected in both environments and explained 10.7% of the PVE at ML and 10.1% at MT. The other two QTL, a major (*SSCE03)* and a minor one (*SSCE11)*, were spotted only in MT, explaining the 14.75 and 7.8% of PVE. The “67/3” allele at *SSCE04* was associated with an increment in soluble solid content, while “305E40” alleles contribute to increase soluble solid content at *SSCE03* and *SSCE11*.

### QTL determining fruit glycoalkaloid traits in eggplant

#### Solasonine (SS)

No QTL exceeding the minimum threshold was identified for this trait.

#### Solamargine (SM)

*SME06* was the only QTL detected in ML, with 13.9% of PVE explained. The allele responsible for increased solamargine content derived from “67/3.”

### QTL determining sugars and acids content

#### Fructose (Fru)

A single major QTL (*FruE04*) was identified only in MT, explaining 10.7% of the PVE. The “67/3” allele was associated with an increase of fructose content.

#### Glucose (Glc)

A single major QTL (*GlcE04*) was identified only in MT, near *FruE04*, explaining 10.7% of the PVE. The “67/3” allele was associated with an increase of glucose content.

#### Sucrose (Suc)

No QTL exceeding the minimum threshold was identified for this trait.

#### Quinic acid (QA)

A major (*QAE01*) and a minor (*QAE09)* QTL were identified only in MT location, explaining 16.4 and 8.9% of the PVE, respectively. The “67/3” allele was associated with an increase in quinic acid.

#### Citric and oxalic acid (CA and OA)

No QTL exceeding the minimum threshold were identified for these traits.

#### Shikimic acid (SA)

A major (*SAE02*) and a minor (*SAE09)* QTL were identified only in ML, explaining 12.1% and 8.2% of the PVE, respectively. The positive alleles derived from “305E40.”

#### Chlorogenic acid (CGA)

*Two* conserved QTL in both the environments were identified. The QTL *CGAE06* was major in MT and minor in ML, explaining 15.4 and 7% of the PVE, respectively. QTL *CGAE04* was minor in both environments and explained 7.3 and 9.5% of the PVE in ML and MT, respectively. All the positive alleles derived from “305E40.”

### Epistasis

Epistatic interactions were evaluated by considering the two sites as independent replicates (Figure 2). In ML, epistatic interaction was observed for *Undcal*: a pair of previously detected QTL (UndcalE05.ML^*^ and UndcallE10.ML^*^) displayed a significant level of additive x additive, additive x dominant and dominant x additive epistasis, with an individual variance of 0.7, 0.5, and 1.3%, respectively. In MT, epistatic interactions were observed for *Frucol, Undcal*, and *D3R*. For *Frucol*, an already identified QTL FrucolE05.MT^*^ together with a *de novo* QTL (*epFrucolE09.MT*) displayed a significant degree of additive x additive and dominant x additive epistasis, explaining 3.4 and 0.4% of the PVE. The previously detected UndcalE05.MT^*^ and UndcallE10.MT^*^ showed additive × additive, additive × dominant, dominant × additive, and dominant × dominant epistasis, explaining 0.5, 0.7, 1.6, and 0.7% of the PVE. In addition, the previously identified UndcallE10.MT^*^ showed additive × dominant and dominant × dominant epistatic interactions with a *de novo* QTL (*epUndcalE05.MT*), explaining 0.3 and 1% of PVE. Finally the brand new *epD3RE02.MT* QTL epistatically interact (additive × additive, additive × dominant, dominant × additive, and dominant × dominant) with the already identified *D3RE05.MT* region, explain 4.5, 2.4, 1.4, and 0.8% of the PVE. The combined site analysis showed that none of the additive effect × site or dominance effect × site interactions were statistically significant at *p* < 0.05.

### Candidate genes identification based on orthology with tomato

The tomato QTL identified by Fulton et al. (2002) affecting the content in fructose (*fru4.1*), glucose (*glu4.1*), and °Brix (*brx4.1*), as well as the QTL *fru-4I, glu-4I*, and *brx-4I* reported by Causse et al. (2004) lie in a region which is syntenic to E04 region in which clustered the eggplant QTL controlling fructose, glucose and soluble solid content (*FruE04.MT, SSCE04.ML, SSCE04.MT, GlcE04.MT*).

The eggplant *QAE01.MT* and *QAE09.MT* regions are syntenic to the IL1-1 and IL9-3 reported to contain metabolic QTL for quinate. Similarly, the *CGAE04* and *CGAE06* QTL are syntenic to the IL4-1 and IL 6-3 containing QTL for the quinate (Schauer et al., 2006).

A blastX search of the NCBI non-redundant protein database carried out for the marker loci linked to the QTL, failed to highlight any known genes or transcription factors involved in anthocyanin, sugar, acid, and glycoalkaloid pathways.

To identify candidate genes underlying the eggplant QTL identified in the present study, we investigated (the tomato syntenic regions sequence (build 2.50; http://solgenomics.net/organism/Solanum_lycopersicum/genome) to search for genes and transcription factors putatively involved in the control of the traits in study. Among the tomato genes/transcription factors involved in anthocyanin biosynthesis, the gene encoding UDP glucose anthocyanidin 3-0 glucosyltansferase (3GT), *an2* and *ant1* are all located on chromosome T10, proximal to the E10 region containing 15158_PstI_L379 (linked to QTL for *Undcal* and *Pncc*). The anthocyanin synthesis-associated enzyme UDP glucose anthocyanidin 5–0 glucosyltransferase is located on T12, in a region distal to E05 region containing 3311_PstI_l361 (linked to QTL for *Frucol, Undcol, D3R*, and *Nas*).

Among the genes/transcription factors involved in the fructose/glucose pathways, we identified a UDP-glucosyltransferase (Solyc04g080010.2.1) and a Glycosyltransferase family 77 protein (Solyc04g080080) in the IL4-4 region (syntenic to *FruE04.MT, SSCE04.ML, SSCE04.MT, GlcE04.MT*), which was reported as containing QTL controlling the fructose and glucose content.

## Discussion

### Phenotyping, QTL mapping, and clustering

Nowadays, consumers are aware of the beneficial effect of consuming fruits and vegetables as many evidences now exist concerning their protective effect against a number of diseases. However, consumers do not choose their foods exclusively for the nutrients they provide but also on the basis of their taste, texture, and appearance (Causse et al., 2010). In recent years a wide number of studies have been focused in assessing the content in phytonutrients of vegetables (Daunay et al., 2001; Gupta and Prakash, 2014) but a limited number of studies have been carried out in identifying the genetic basis influencing their content and their interaction with the environment.

Within the Solanaceae family, efforts toward the characterization of QTL controlling the biochemical properties and nutritional value of the berries have been mainly carried out in tomato (Fulton et al., 2002; Causse et al., 2004; Sacco et al., 2013), while in potato the genetic basis of tuber quality and glycoalkaloids content (Bradshaw et al., 2008; D'hoop et al., 2008; Sørensen et al., 2008; Valcarcel et al., 2014) have been investigated. In eggplant, studies have been focused on polyphenols, anthocyanins, chlorogenic acid (Stommel and Whitaker, 2003; Mennella et al., 2010) as well as glycoalkaloids content (Aubert et al., 1989; Sánchez-Mata et al., 2010). However, QTL controlling basic qualitative traits, such as °Brix, simple sugars, organic acids, DM, and health-related metabolites have not been so far characterized, in spite of their key role in breeding programs for developing eggplants with improved content of phenolics, and other health-related promoting compounds.

Evidences that season, environment and genotype strongly affect eggplant fruit composition of carbohydrates and starch, vitamin C and phenolics has been recently ascertained (San José et al., 2014; Stommel et al., 2015). Similar results were also reported for dry matter, proteins, total phenolics, and mineral contents (Raigón et al., 2010) as well as solasonine, D3R and solamargine (Mennella et al., 2010). In the present work, the high $h_{BS}^{2}$ value associated with the traits recorded along with the correlation between the two sites highlight that the environment played a moderate influence on the phenotypic outcome of the segregating F_2_ in study. The *h*${}_{BS}^{2}$ values were higher than 60% for all the traits (Table 3) with the exception of SSC in both locations (0.41% in ML and 0.43% in MT) and DM in MT (0.51%). Collard et al. (2005) suggested that a QTL should only be classified as “major” if it can account for more than 10% of the PVE. A more stringent definition of “major” implies the conservation of a QTL in multiple seasons/locations (Li et al., 2001; Lindhout, 2002; Pilet-Nayel et al., 2002). Among the 14 QTL discovered in ML and the 15 in MT, at least one major QTL per each trait in study was identified. The less and the most convincing LOD scores associated with these QTL were 3.85 (*SSCE04.ML* and *GlcE04.MT*) and 72.35 (*UndcalE10.MT*) respectively. The explained PVE varied from ~10% (*SSCE04.MT*) up to ~86.9% (*PnccE10.ML*) and most of the identified QTL were stable across the two environments making them useful in the context of marker-assisted selection. On the other side the identification of QTL in just one environment (i.e., *DME02.ML* or *FruE04.MT*), supported the strong environment effect on some metabolic traits in eggplant, as described above.

Finally, some traits were QTL orphan: presumably, the genetic variance between the parents and/or the limited variation in the mapping population were not sufficient to dissect the genetic basis of these traits (Lander and Botstein, 1989).

The genetic basis of anthocyanin synthesis and accumulation has been widely studied in the Solanaceae (van Eck et al., 1993, 1994; Chaim et al., 2003; Borovsky et al., 2004; De Jong et al., 2004; Bovy et al., 2007; Gonzali et al., 2009). The genetic control of their accumulation and distribution in eggplant was for long time thought to be complex, involving at least three major and five minor loci with assumed epistatic interactions and/or pleiotropic effects (Tatebe, 1939; Tigchelaar et al., 1968). Recently, QTL-related studies using a family based and association mapping approach allowed to furtherly clarify the genetic basis of anthocyanin distribution in eggplant tissues and organs as well as to identify its syntenic relationships with tomato (Barchi et al., 2012; Ge et al., 2013; Cericola et al., 2014). The QTL cluster identified on chromosome E05, which controls *Frucol, Undcal*, the content of the anthocyanins *D3R* and *Nas* responsible of the peel color together with the detected inter-trait correlations, suggests the presence of a putative pleiotropic locus. Using the same mapping population, Barchi et al. (2012) identified QTL controlling several anthocyanin-related traits and the color of the corolla in the same chromosome region, while Ge et al. (2013) and Cericola et al. (2014) identified similar QTL in the distal portion of E05. Our data confirm that the bottom part of the E05 is clearly involved in anthocyanin production and distribution among the eggplant tissues and organ. A second QTL cluster for *Pncc* and *Undcal* was identified on E10, and is collinear with the one previously identified in several plant tissues by using the same F_2_ mapping population (Barchi et al., 2012) as well as with GWAS approach (Cericola et al., 2014). This chromosome region may be ascribable to the same QTL previously identified by Doganlar et al. (2002b) and involved in anthocyanin accumulation.

The QTL located on E5 and E10 are possibly involved in two different aspects of the anthocyanic determination and distribution in eggplant.The QTL on E05 is involved both in the alternative production of the nasunin or D3R and it is responsible of the fruit and corolla color. This hypothesis is supported by the following considerations: (i) the two QTL co-localize at the same molecular marker; (ii) nasunin completely replaced D3R in the parental line “67/3” and in the hybrid, which suggest that it originates from an enzymatic modification of D3R (Kroon et al., 1994; Fujiwara et al., 1997; Yonekura-Sakakibara et al., 2000; Ichiyanagi et al., 2005; Azuma et al., 2008). However, no genetic locus encoding the 5-5-O-glucosyltransferase gene or of a cumaryltransferase has been to date identified. Conversely, the QTL on chromosome E10 appears mainly involved in the color determination of the peel next to the calyx (*Pncc*) and in anthocyanin intensity in all the other tissues. The joint effect of both E05 and E10 QTL influence *Undcal*, alluding to an interaction between genes influencing both *D3R*/*Nas* and *Pncc* in controlling this trait.

The steroidal glycoalkaloids are distinctive Solanaceae secondary metabolites; they are warehoused in all tissues, including fruits (tomato and eggplant) and tubers (potato) (Friedman, 2002, 2006; Kozukue et al., 2008; Mennella et al., 2010; Sawai et al., 2014) and provide the plant with a chemical barrier against a broad range of pathogens (Chan and Tam, 1985; Hoagland, 2009). Their biosynthesis starts from cholesterol, which is converted firstly in steroidal alkaloids (SAs) and subsequently decorated with various sugar moieties to generate steroidal glycoalkaloids (SGAs, Itkin et al., 2013), and require genes encoding uridine 5′-diphosphate (UDP)–glycosyltransferases (UGTs), like GAME1 in tomato (Itkin et al., 2011) or SGT1 in potato (McCue et al., 2005). Recently, Sawai et al. (2014) identified St SSR2 as a key enzyme in the biosynthesis of toxic SGAs derived from cholesterol in potato. SGAs deeply influence the organoleptic properties of the flesh, by conferring a typical bitter taste (Aubert et al., 1989; Sánchez-Mata et al., 2010) and are potentially toxic to animals and humans, although some recent studies support also their anti-cancer and anti-microbial properties (Blankemeyer et al., 1997; Friedman et al., 2009; Milner et al., 2011). Reduction of the SGAs eggplant content represent a key breeding aspect for the crop improvement. The QTL analyses conducted over the two main eggplant SGAs, solasonine and solamargine, revealed the presence of one major QTL, *SME06.ML* only in ML. The blastX results did not identify any putative gene.

Sugars and acids, involved in soluble solid content and dry matter, are key quality components of the fruit quality and influence its nutritional values and organoleptic properties. QTL related for sugar and acids content were found in only one of the sites in study (ML for *SA* and MT for *Fru, Glc* and *QA*) as confirmed by their medium-low inter-site correlations. This finding suggests that environment influenced these traits although they had a high heritability, good inter-traits correlations (*Fru* with *Glc* and *SA* with *QA*) and a PV >10%; similar results were reported by San Josè et al. (2014). This phenomenon may also partially explain why QTL for sucrose, oxalic acid and citric acid content were not identified.

The chlorogenic acid in eggplant berries plays a well-documented anti-oxidant activity. The chlorogenic synthesis pathway in Solanaceae is quite well-known (Clé et al., 2008) and the sequences of the six genes codifying for the enzymes involved in this pathway have been studied. In a recent study, most of the genes involved in the polyphenols and chlorogenic acid biosynthetic pathway were positioned in an interspecific map by using a candidate gene approach (Gramazio et al., 2014). It has been demonstrated that the growing environment and its interaction with the genotype influences phenolic acid content; nonetheless, a reduced variability in phenolics may be obtained through selection for stability (Stommel et al., 2015). The two *CGA* QTL, identified in both locations, however did not contain any putative gene involved in the chlorogenic acid pathway. Thus, the genetic basis of the CGA related trait, as well as that of glycoalkaloids, deserve to be further investigated (e.g., by means of a candidate gene approach on the basis of the already identified genes in tomato and potato).

### Parental alleles, transgressive segregation, and epistasis

For some traits the parental origin of the QTL alleles reflected the performance of the parents; thus, for example, the positive alleles at *D3R* and *SA* were inherited from “305E40” while *Frucol, Nas* and *Glc* derived from “67/3.” On the contrary, *Pncc* and *SSC* were traits increased by positive alleles coming from both the parents. Finally, positive alleles for *CGA* and *QA* derived from the less performant parent.

Transgressive segregation, which derives from the combination of alleles from both parents having effect on the same direction (deVicente and Tanksley, 1993), was found for several traits, and in most cases displayed the same transgressive trend in both the environment (as *Frucol*, or *Undcal*). On the contrary, some traits (e.g., *SS* and *OA*), showed a different transgressive trend due to the different performances of the parents at the two sites. This phenomenon is common in QTL analysis and already observed in eggplant (Barchi et al., 2012; Portis et al., 2014).

In addition to a conserved epistatic interaction for *Undcal* between the two sites, a number of environmental-specific examples of epistasis were noticed, none of them explained a substantial proportion of the PV (0.3–4.5%). Such phenomenon previously detected in the same mapping population (Barchi et al., 2012; Portis et al., 2014) may be explained by invoking interference from other QTL in the background (Al-sane et al., 2011). Although the analysis carried out with QTLNetwork 2.1 (Yang et al., 2007) on the combined data set produced no significant QTL x Environment interactions, some QTL identified with the MQM were location-specific, for this reason we cannot rule out the presence of QTL x Environment interaction.

### Synteny and putative orthologous QTL

Tomato represents a model species in the Solanaceae family and many studies have been carried out to better understand the genetic basis underlying yield (Brix and SSC), fruit flavor, and taste (sensory quality, glycoalkaloids) by means of deep metabolic profiling and candidate gene survey (Fridman et al., 2000; Fulton et al., 2002; Causse et al., 2004; Schauer et al., 2006; Bermúdez et al., 2008; Itkin et al., 2011; Iijima et al., 2013).

The extensive synteny between the tomato and eggplant genomes enables genetic inferences to be made in eggplant exploiting the much greater knowledge of tomato genome (Wu et al., 2009; Barchi et al., 2012; Fukuoka et al., 2012; Tomato genome consortium, 2012). Furthermore, the availability of the eggplant genome (Hirakawa et al., 2014) confirmed the synteny level existing between eggplant and tomato.

Schauer et al. (2006) conducted a deep metabolic QTL analysis using the tomato ILs population developed by Liu and Zamir (1999). The IL4-4 was reported as containing QTL controlling the fructose and glucose content; in the corresponding eggplant genomic region we identified two QTL involved in the accumulation of fructose and glucose (*FruE04.MT* and *GlcE04.MT*). In this tomato region, a glycosyltransferase family GT8 protein, an UDP-glucosyltransferase, and a glycosyltransferase family 77 protein were identified, which might be involved in the sugar pathway. The eggplant *QAE01.MT* and *QAE09.MT* QTL are syntenic to ILs 1-1/1-1-3 and IL9.3 but no putative genes involved in the quinic acid pathway have been identified. Finally, the two QTL identified for CGA lie in tomato syntenic regions of the ILs 4-1 and 6-3, involved in quinic acid accumulation. The search for genes involved in chlorogenic pathway did not highlight any putative gene, although the CGAs pathway is well-known and eggplant and tomato genes have been identified in the work of Hirakawa et al. (2014).

In summary, the QTL here detected, provide important information on the genomic region associated to fruit biochemical and phenotypical properties in eggplant which may be usefully exploited in view of breeding programs aimed at releasing of new eggplant varieties with superior commercial, organoleptic and compositional characteristics.

## Author contributions

GLR, SL, NA, RL, GM conceived and designed the research; LT, LB, RL, ErP, GF, MF, AD, VP, VL, LS, LP, TS, NA, GM performed fruit phenotypical and biochemical characterization; LT, LB and EzP analyzed the data; LT, LB, GLR wrote the manuscript. All authors read and approved the manuscript.

## Funding

This research was partially supported by the Italian Ministry of Agriculture in the framework of the project “IT-IL-NUTRISOL ITalian-IsraeLi initiative for the NUTRItional improvement of SOLanaceus crops” (DM 27723/7303/11).

### Conflict of interest statement

The authors declare that the research was conducted in the absence of any commercial or financial relationships that could be construed as a potential conflict of interest. The reviewer MP and Handling Editor declared their shared affiliation, and the Handling Editor states that the process nevertheless met the standards of a fair and objective review.
