# Supplementary material for: Mapping Quantitative Trait Loci Affecting Biochemical and Morphological Fruit Properties in Eggplant (Solanum melongena L.)
Source: Front Plant Sci. 2016 Mar 4;7:256. doi: 10.3389/fpls.2016.00256 (PMC4777957; doi:10.3389/fpls.2016.00256)
Supplement: Supplementary file 1 [file Table1.DOCX]

Supplementary Table 1. List of the traits (and unit of measurement) analyzed in a preliminary study for the four ripening stages A (immature), B-(almost commercial ripe), B (commercial ripe) and C (physiological ripe). For each stage, means ± standard deviations (SD) and ssignificant mean difference among parental values (T test) is reported (*p<0.05). Stage A was chosen as reference for the flesh biochemical characterization as it was the stage in which the parental lines showed the highest significant differences with regard to the content of the majority of the compounds in study

| Trait | measure unit | | A | | | | B- | | | | B | | | | C | | | |
| --- | --- | --- | --- | --- | --- | --- | --- | --- | --- | --- | --- | --- | --- | --- | --- | --- | --- | --- |
|  |  | |  | | | |  | | | |  | | | |  | | | |
|  |  | | 67-3 | 305 E 40 | P(F<=f) |  | 67-3 | 305 E 40 | P(F<=f) |  | 67-3 | 305 E 40 | P(F<=f) |  | 67-3 | 305 E 40 | P(F<=f) |  |
|  |  |  |  |  |  |  |  |  |  |  |  |  |  |  |  |  |  |  |
| Chlorogenic acid | µmol/100g dw |  | 4681.09±967.27 | 8092.14±159.84 | 0.0004 | * | 3024.65±57.48 | 2941.232±293.25 | 0.5968 |  | 2587.84±907.95 | 2581.90±324.41 | 0.9906 |  | 1259.57±116.99 | 1541.52±12.52 | 0.0030 | * |
| Solamargin | µmol/100g dw |  | 4.25±2.04 | 11.15±1.15 | 0.0011 | * | 1.30±0.13 | 3.45±0.66 | 0.0014 | * | 0.82±0.05 | 0.75±0.14 | 0.3981 |  | 14.28±3.80 | 23.74±7.77 | 0.0713 |  |
| Solasonine | µmol/100g dw |  | 38.27±2.47 | 12.89±1.07 | 1.45E-06 | * | 10.74±1.35 | 23.41±3.71 | 0.0007 | * | 10.83±0.90 | 25.39±2.44 | 3.02E-05 | * | 11.86±8.89 | 59.47±6.46 | 0.0001 | * |
| Nasunin | mg/100 g peel dw |  | 243.58±26.82 | 0 | 1.79E-06 | * | 273.01±10.50 | 0 | 3.40E-09 | * | 313.78±16.20 | 0 | 1.98E-08 | * | 146.41±5.27 | 0 | 2.28E-09 | * |
| D3R | mg/100 g peel dw |  | 0 | 701.81±66.66 | 7.47E-07 | * | 0 | 1070.97±84.97 | 2.40E-07 | * | 0 | 535.67±67.37 | 3.02E-06 | * | 0 | 102.03±11.38 | 1.94E-06 | * |
| Soluble solids | % d.w. |  | 60.22±1.39 | 39.11±2.78 | 9.27E-06 | * | 58.89±2.04 | 51.33±0.67 | 0.0004 | * | 60.00±1.15 | 50.44±2.14 | 0.0002 | * | 55.11±2.34 | 48.67±1.33 | 0.0030 | * |
| Dry matter | % d.w. |  | 9.32±0.75 | 7.63±0.39 | 0.0070 | * | 8.35±0.87 | 8.91±1.31 | 0.5727 |  | n.d | n.d |  |  | n.d | n.d |  |  |
| Sucrose | mg/ 100 mg dw |  | 942.62±294.94 | 1345.08±134.41 | 0.0476 | * | 1031.02±726.67 | 792.90±365.51 | 0.6388 |  | n.d | n.d |  |  | n.d | n.d |  |  |
| Glucose | mg/ 100 mg dw |  | 19827.16±3851.37 | 17128.97±3441.23 | 0.2296 |  | 22899.96±2320.31 | 20159.14±1899.44 | 0.1886 |  | n.d | n.d |  |  | n.d | n.d |  |  |
| Fructose | mg/ 100 mg dw |  | 18820.68±4842.37 | 13746.30±2425.75 | 0.0446 | * | 22899.96±2320.31 | 19099.89±2813.82 | 0.1454 |  | n.d | n.d |  |  | n.d | n.d |  |  |
| Ossalic acid | mg/ 100 mg dw |  | 4729.03±2038.22 | 3495.02±1652.18 | 0.4610 |  | 2946.56±556.15 | 2982.43±1504.83 | 0.9710 |  | n.d | n.d |  |  | n.d | n.d |  |  |
| Quinic acid | mg/ 100 mg dw |  | 1756.63±491.07 | 4100.92±1167.87 | 0.0011 | * | 1248.07±150.08 | 1492.08±433.42 | 0.4090 |  | n.d | n.d |  |  | n.d | n.d |  |  |
| Shikimic acid | mg/ 100 mg dw |  | 597.98±57.49 | 1137.30±192.52 | 0.0001 | * | 415.47±149.35 | 565.25±104.27 | 0.2275 |  | n.d | n.d |  |  | n.d | n.d |  |  |
| Citric acid | mg/ 100 mg dw |  | 453.30±311.65 | 868.50±242.32 | 0.0276 | * | 672.33±165.98 | 260.64±95.81 | 0.0205 | * | n.d | n.d |  |  | n.d | n.d |  |  |
| Malic acid | mg/ 100 mg dw |  | 1433.84±769.19 | 242.33±127.16 | 0.0502 |  | 1308.38±168.73 | 969.65±231.76 | 0.1101 |  | n.d | n.d |  |  | n.d | n.d |  |  |
| Total polyphenols | mg/100 mg dw |  | 2180.2±199.9 | 2915±137.3 | 0.0063 | * | 1312.0±192.2 | 1461.80±33.6 | 0.2543 |  | 1378.6±33.6 | 1181.6±52.0 | 0.0053 | * | 1117.9±165.2 | 1098.4±30.0 | 0.8511 |  |
| PPO activity | (U/100 mg dw) |  | 833.3±92.9 | 766.7±130.3 | 0.4372 |  | 594.4±19.2 | 672.1±34.5 | 0.0077 | * | 544.4±19.2 | 461.1±69.4 | 0.0654 |  | 177.8±9.7 | 372.2±50.7 | 0.0003 | * |
| Peroxyl | eqCA/100 g dw |  | 1.61±0.42 | 1.53±0.03 | 0.3850 |  | 1.88±0.02 | 1.40±0.06 | 0.0001 | * | 1.52±0.19 | 1.30±0.06 | 0.0633 |  | 1.37±0.06 | 1.29±0.34 | 0.3608 |  |
| Superoxide | eqCA/100 g dw |  | 4.44±0.71 | 9.07±0.80 | 0.0017 | * | 3.88±1.37 | 3.22±0.53 | 0.4798 |  | 2.66±1.01 | 3.15±0.96 | 0.5736 |  | 1.89±0.45 | 2.94±0.85 | 0.1319 |  |
| Idroxyl | eqCA/100 g dw |  | 13.00±4.20 | 8.28±6.47 | 0.3492 |  | 7.06±1.02 | 9.26±1.64 | 0.1200 |  | 13.08±7.38 | 5.17±1.18 | 0.1405 |  | 7.45±3.53 | 6.77±3.82 | 0.8311 |  |
| Acidity | meq/100g dw |  | 34.9±13.4 | 41.7±8.6 | 0.4289 |  | 26.6±0.6 | 62.0±34.9 | 0.0885 |  | 32.7±4.6 | 25.6±2.6 | 0.0362 | * | 28.8±4.2 | 42.9±4.2 | 0.0031 | * |
